# Supplementary material for: The Use of Calcaneal Quantitative Ultrasound as a Bone Health Screening Tool Amongst People Living with HIV and Taking Tenofovir-Based Antiretroviral Therapy: A Pilot Study
Source: Biomedicines. 2025 Nov 21;13(12):2847. doi: 10.3390/biomedicines13122847 (PMC12730778; doi:10.3390/biomedicines13122847)
Supplement: Supplementary file 1 [file biomedicines-13-02847-s001.zip › biomedicines-3982491-supplementary.pdf]

Table S1. Correlation between the duration of ART treatment and bone health indices.

		SOS	BUA	OI	QUS T-Score	Femoral Neck BMD	Femoral Neck T-Score	Lumbar Spine BMD	Lumbar Spine T-Score
Duration of ART	ρ	0.056	0.114	0.127	0.126	−0.062	−0.077	0.126	0.133
	p	0.692	0.422	0.371	0.375	0.664	0.587	0.373	0.346
BMI	r	0.082	−0.023	0.046	0.051	0.473	0.489	0.281	0.283
	p	0.564	0.874	0.745	0.722	<0.001	<0.001	0.044	0.042

Abbreviations: ρ, Spearman's regression coefficient; ART, antiretroviral therapy; BMD, bone mineral density; BUA, broadband attenuation of sound; OI, osteoporotic indices; QUS, quantitative ultrasound; r, Pearson's regression coefficient; SOS, speed of sound.
